# Energy-Efficient Medium Access Control Protocols for Cognitive Radio Sensor Networks: A Comparative Survey

**DOI:** 10.3390/s18113781

**Published:** 2018-11-05

**Authors:** Subash Luitel, Sangman Moh

**Affiliations:** Department of Computer Engineering, Chosun University, 309 Pilmun-daero, Dong-gu, Gwangju 61452, Korea; karyon25@gmail.com

**Keywords:** wireless sensor network, cognitive radio, cognitive radio sensor network, medium access control, network protocol, energy efficiency, network lifetime

## Abstract

The increase of application areas in wireless sensor networks demands novel solutions in terms of energy consumption and radio frequency management. Cognitive radio sensor networks (CRSNs) are key for ensuring efficient spectrum management, by making it possible to use the unused licensed frequency spectrum together with the unlicensed frequency spectrum. Sensor nodes powered by energy-constrained batteries necessarily require energy-efficient protocols at the routing and medium access control (MAC) layers. In CRSNs, energy efficiency is more important because the sensor nodes consume additional energy for spectrum sensing and management. To the best of authors’ knowledge, there is no survey on “energy-efficient” MAC protocols for CRSNs in the literature, even though a conceptual review on MAC protocols for CRSNs was presented at a conference recently. In this paper, energy-efficient MAC protocols for CRSNs are extensively surveyed and qualitatively compared. Open issues, and research challenges in the design of MAC protocols for CRSNs, are also discussed.

## 1. Introduction

With recent advancements in micro-electromechanical systems (MEMS), the development of smart sensor nodes of both small size and low cost has become increasingly popular [1,2]. Wireless sensor networks (WSN) consist of multiple sensor nodes and are used for various applications. In general, WSN applications operate on unlicensed industrial, scientific, and medical (ISM) bands that are becoming more overcrowded every day, since these bands are shared with other wireless applications. Meanwhile, the licensed bands are not efficiently used, thus leading to a serious imbalance in radio spectrum use. As a result, the development of wireless applications requires not only efficient use of available spectrum, but also effective spectrum management [3]. In this context, the concept of cognitive radio (CR) was introduced [4,5] to utilize the available licensed spectrum by exploiting its cognitive capabilities and reconfigure ability features [6]. This enabled unlicensed secondary users (SUs) to use part of the underutilized licensed spectrum without interfering with the activities of licensed primary users (PUs).

A cognitive radio sensor network (CRSN) is a WSN with CR capabilities. In addition to the opportunistic use of the licensed spectrum, SUs can utilize the unlicensed ISM bands as well without impeding communication among PUs. Therefore, a CRSN requires the fundamental features of CR networks, which include spectrum sensing, spectrum decision, channel handoff, and so on [7].

To support opportunistic use of the licensed spectrum, much more attention should be paid to the design of medium access control (MAC) protocols in CRSNs. MAC protocols play a pivotal role in handling the key activities in CRSNs, from resource allocation to channel sensing, spectrum decisions, spectrum mobility and spectrum sharing. Like in other WSNs, energy efficiency is the most important factor in CRSNs, because they are powered by batteries and possess the inherent characteristics of WSNs. In fact, CRSNs can consume even more energy than other WSNs, because of the additional necessary tasks of channel sensing and switching.

As in WSNs, energy efficiency is one of the most important design considerations in CRSNs, because it directly affects network lifetime. Hence, different approaches have been proposed to make sure that battery use is minimized to significantly prolong network lifetime. One of the most effective methods proposed is the use of an energy-efficient MAC protocol that does not degrade performance. Several energy-efficient protocols for CRSNs have been designed and implemented to prolong network lifetime with reasonable success so far. A conceptual review on MAC protocols for CRSNs was presented [8]. On the other hand, a review on energy-efficient MAC protocols for cognitive radio ad hoc networks was presented [9]. However, to the best of authors’ knowledge, there is no survey on “energy-efficient” MAC protocols designed for CRSNs in the literature. In this paper, we present a survey of energy-efficient MAC protocols for CRSNs, and then compare them qualitatively. Some open issues and research challenges are also discussed with respect to the design of MAC protocols for CRSNs.

The rest of this paper is organized as follows. The major features of CRSNs are overviewed in Section 2; the system’s architecture, its competitive advantages over other WSNs, and different application areas are summarized. In Section 3, energy efficiency in the design of MAC protocols for CRSNs is addressed. In Section 4, existing energy-efficient MAC protocols for CRSNs are reviewed one by one. In Section 5, they are qualitatively compared with each other. In Section 6, open issues and research challenges are discussed. Finally, this paper is concluded in Section 7.

## 2. Major Features of CRSNs

In this section, the main features of CRSNs are briefly summarized in order for readers to acquire relevant background knowledge for this paper and to enable them to easily follow the remaining sections. In short, the system architecture, its competitive advantages over other WSNs, and various application areas are reviewed.

### 2.1. System Architecture

**Figure 1:**
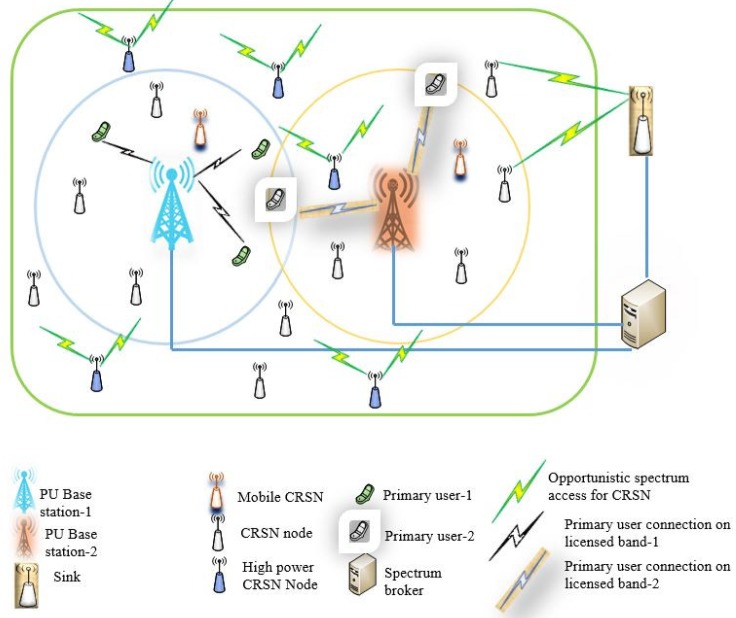
Typical cognitive radio sensor network (CRSN) architecture.

Figure 1 shows the typical architecture of CRSNs in which the readings of sensor nodes are transmitted to the sink in such a way that the sensor nodes transmit their reading to the next hop in an opportunistic manner and, eventually, to the sink [7]. The sink may or may not have cognitive radio capabilities. The sensor nodes may also be involved in the exchange of control messages for group formation, spectrum allocation, and route determination.

The cognitive radio transceiver enables sensor nodes to dynamically adapt different communication parameters, such as carrier frequency, transmission power, and modulation. The CRSN nodes are also constrained in power and memory, like other WSN nodes. CRSNs may have different topologies for different applications, which are discussed below. It is imperative to consider the specific network topology to be used while designing the protocol. This is so because the mechanisms used in the protocol for one topology to enhance the overall communication performance and reduce energy consumption may not be adequate for other topologies.

#### 2.1.1. Ad Hoc CRSNs

Ad hoc CRSNs have an infrastructure less topology in which the readings of nodes are sent to the sink in an ad-hoc manner in multiple hops. In this topology, the sensor node itself may be involved in spectrum sensing or this sensing may be collaborative in a distributed way. This type of topology results in low communication overhead for control data; however, it suffers from the hidden terminal problem, which causes inaccuracy in spectrum sensing and can result in performance degradation in PU communication.

#### 2.1.2. Clustered CRSNs

Designating a common control channel (CCC) for exchanging control data is an essential task in CRSNs. Control data includes neighbor discovery, spectrum sensing results, spectrum allocation, and maintenance information. However, for an entire network, it is not always possible to find such a CCC. According to previous research [10], we can find such a CCC in a specific restricted locality, exploiting the spatial correlation of channel availability. The clustering topology is a promising topology because we can select a CCC to more effectively enhance the dynamic spectrum management process. In addition, the cluster head in a network with clustered topology can carry out local bargaining, as well as collection and dissemination of information about spectrum availability.

#### 2.1.3. Heterogeneous and Hierarchical CRSNs

A CRSN including some high-power relay nodes with longer transmission ranges forms a heterogeneous and multi-layer hierarchical topology. Such a network consists of sensor nodes, high-power relay nodes, and a sink. However, the deployment of sensor nodes and high-power relay nodes increases communication overhead, due to the hierarchical coordination process.

#### 2.1.4. Mobile CRSNs

A mobile CRSN possesses a dynamic topology, caused by the movement of any architectural element of the CRSN. For instance, CRSN nodes may move from one position to another according to the application area for which they are deployed. Mobility-aware spectrum techniques are thus indispensable for this kind of topology.

### 2.2. Competitive Advantages over Other WSNs

Dynamic spectrum access capacity is a key point [7] that makes CRSNs a more promising technology in comparison to the conventional WSNs. The following network capabilities are effectively facilitated by CRSNs.

#### 2.2.1. Dynamic Spectrum Access

In general, conventional WSNs use fixed spectrum bands in the same range as other wireless communication technologies. For example, IEEE802.11 wireless local area network (WLAN) hotspots, personal digital assistants (PDAs) and Bluetooth devices heavily use the same unlicensed bands. In addition, a spectrum lease for a licensed band would be expensive. Therefore, the dynamic spectrum access feature in CRSNs helps to cooperate with existing licensed users and use the licensed spectrum opportunistically

#### 2.2.2. Opportunistic Channel Usage for Bursty Traffic

The traffic produced in WSNs is bursty in nature and densely deployed sensors increase the probability of collisions, resulting in performance degradation and significant power consumption. All the nodes may try to simultaneously capture the channel to establish their communication link, and CRSN makes it easy for the nodes to use multiple available channels opportunistically to reduce collision rates.

#### 2.2.3. Adaptability to Mitigate Energy Consumption

Wireless channels are time varying in nature, resulting in packet losses and retransmissions. This causes a higher consumption of energy. CRSNs have the capability of adapting to various channel conditions by changing their operational parameters, which ensures higher transmission efficiency by reducing the required reception and transmission energy.

#### 2.2.4. Access to Multiple Channels under Different Spectrum Regulations

Since different countries or different regions within a country may allocate spectrum bands differently because of spectrum regulations, traditional sensor nodes are not always operable in different locations since there may be no matching spectrum available. However, CRSNs are capable of changing their frequency channel and hence help to mitigate this problem.

#### 2.2.5. Overlaid Deployment of Multiple Concurrent WSNs

Due to the dynamic spectrum management capability of CRSNs, the coexistence of spatially overlapping sensor networks is possible. This is beneficial in terms of resource utilization and communication performance.

### 2.3. Different Application Areas

Because of several advantages of CRSNs over conventional WSNs, the existing application areas for WSNs can be enhanced with better communication performance by deploying CRSNs. Different application areas use different sensors, such as seismic, thermal, visual, infrared, and acoustic radar sensors. Currently, sensor networks are heavily deployed in various sectors, such as in environmental, health, military, smart grid, transportation, and vehicular applications, as well as in other commercial areas in which CRSNs can be effectively exploited.

#### 2.3.1. Environmental Applications

Sensor nodes are heavily deployed in environmental applications to facilitate flood and forest fire detection and measure the amount of pesticides in drinking water, pollution levels, etc. [11,12,13,14].

#### 2.3.2. Health Applications

Health applications include monitoring vital signs, such as heart rate, blood pressure, sugar level, etc. The physiological data collected by sensor networks may be helpful for future researches seeking to develop appropriate medication by analyzing the behavior of existing data and could also help to identify predefined symptoms earlier. The behavior of old people can also be monitored and events, such as falls can be detected using sensor networks. This kind of devices is known as wireless body area networks (WBANs) [15]. Moreover, in hospitals, sensor nodes can be used for tracking the location of doctors and patients. Sensor nodes could also facilitate drug administration in hospitals by reducing the chance of prescribing the wrong medication to a patient, since the information collected from sensor nodes can be stored in a computerized system and would make it easy to identify the exact problem of a patient and, therefore, the exact medicine that particular patient needs [16]. Patients will also benefit from telemedicine [17].

#### 2.3.3. Smart Home Applications

Sensor nodes can be used in many electronic home appliances, such as microwave ovens, vacuum cleaners, rice cookers, and refrigerators, and can be applied in such a way that allows them to interact with each other. They can be controlled as required from other rooms of the house or even remotely from the outside. Generally, the ISM bands are crowded inside houses [18] and CRSNs can contribute to achieve reliable communication in such a crowded spectrum.

#### 2.3.4. Military Applications

Another important application area of sensor nodes is in the military field, where they can be used for surveying the enemy and can assist during targeting. Different types of attacks, including those using nuclear, biological and chemical weapons, can be detected using these sensors, which is very helpful for reducing the number of casualties by devising an immediate plan accordingly. Sensor nodes can be disposed of after their objective is fulfilled without affecting other military applications [19]. The wide-range frequency hand-off capabilities of CRSNs help to avoid jamming signals. In addition, the requirement of a greater bandwidth and smaller communication delay can also be met by CRSNs [20].

#### 2.3.5. Transportation and Vehicular Applications

Wireless access for vehicular environments (WAVE) characterized by the IEEE 1609.4 standard operating in the 5.9 GHz band uses one control channel with six service channels [21]. All the users will contend against each other to capture the channel used for transmission in the 5.9 GHz band. This leads to the requirement of WSNs with cognitive capabilities [22].

#### 2.3.6. Smart Grid

Smart grids are yet another application area, where CRSNs can be deployed in different parts of an electrical grid to enhance smart metering, distributed automation, and so forth [23,24]. Sensor nodes can be embedded in transmission towers, transmission lines, distributed power plants substations, etc.

## 3. Energy Efficiency in the Design of MAC Protocols for CRSNs

In general, sensor nodes deployed in a network may be in six different states. The active modes of a node are the sensing mode, the computational mode, the transmission mode, the receiving mode, and the idle mode, while the sleep mode is the one that consumes least energy. The consumed energy values of different states in a sensor node operating at 3 V were reported in a previous study [25], indicating values of 45 mW in the idle state, 76 mW in the transmit state, 55 mW in the receive state, 19 mW in the sensing state, 22 mW in the computation state, and 2 mW in the sleep state. Therefore, this study clearly shows that the least energy is consumed in sleep mode, which significantly saves energy [26]. Collision and interference, control packet overhead, over-emitting, overhearing, and idle listening are the main sources of energy wastage.

The design of energy efficient protocols, particularly the MAC protocol, which is responsible for handling different radio operations, is based on the aforementioned information. The goal of any energy efficient MAC protocol design for sensor nodes is to reduce the energy wastage parameters and ensure sensor nodes adaptively go into sleep mode, while achieving the required quality of service (QoS) of the network and protecting the incumbent user. The MAC protocols designed for cellular systems are based on the primary goal of achieving a high QoS and bandwidth efficiency, but not high energy efficiency. This is because cellular systems are infrastructure-based systems, thus the base station is always provided with unlimited power and mobile users can charge their devices accordingly. Similarly, the MAC protocols designed for Bluetooth and mobile ad-hoc networks (MANETs) are not applicable to sensor nodes as they focus on infrastructure preservation and QoS provisioning for nodes in mobile conditions.

Therefore, MAC protocols for WSNs with the main goal of increasing energy efficiency to prolong the network lifetime have been proposed, keeping other parameters like latency, throughput and bandwidth utilization as secondary attributes [27]. Some single channel WSN MAC protocols [28,29,30] have been designed by defining high energy efficiency as a major objective. Since these protocols do not operate in a multichannel environment, some multichannel MAC protocols WSNs were proposed, such as McMAC [31] and Y-MAC [32]. Aside from their higher communication performance, multichannel WSNs were found to be more energy efficient, because of their lower number of collisions during transmission. However, even these MAC protocols fail to meet the requirement of CRSNs because of other challenges, such as improving spectrum sensing periods, broadcasting over a network-wide common channel and the requirement of a high-priority access mechanism for the distribution of spectrum sensing and decision results.

The MAC protocols designed for cognitive radio address some of the aforementioned challenges, and [33,34] are examples of cognitive radio MAC protocols. However, they do not meet the objectives of WSNs and hence are not suitable for CRSNs. Since MAC protocols for cognitive radio systems require multiple radios, suffers from idle listening and depend on network-wide synchronization, they have a high energy consumption. In addition, the bursty nature of the traffic in WSNs is not addressed in CR MAC protocols. Thus, the performance of cognitive radio MAC protocols is low in a densely deployed WSN.

As discussed in the previous section, sensor nodes are energy-constrained devices. Charging sensor nodes frequently is not feasible, because of the inconvenient location in which those devices are placed in different applications. In addition to this, the communication provider company Cisco has forecasted that there will be 24 billion connected devices by 2020 [35]. Most end devices will be sensor devices, which indicates that such a large number of devices will definitely require greater sources of energy in addition to more radio spectrum. This statistic encourages researchers to consider energy efficiency during design and before implementing sensor networks in the real field. Communication is the most significant source of energy inefficiency and decreasing its energy budget could extend sensor node lifetime. Moreover, since the MAC protocol is one of the most important parts of the protocol stack in communications and controls radio transmissions, a well-designed CRSN MAC Protocol would ensure a longer lifetime of the sensor nodes.

## 4. Energy-Efficient MAC Protocols for CRSNs

A number of MAC protocols have been designed and developed for CRSNs not only for preserving valuable energy, but also for improving network performance. In this section, existing energy-efficient MAC protocols for CRSNs are presented and discussed.

### 4.1. Energy-Efficient Cognitive Radio MAC Protocol for Battlefield Communications

This is the MAC protocol is aimed at preserving essential energy in sensor nodes deployed to form a cognitive radio sensor network [36]. It exploits the concepts of multichannel communication coupled with time division multiple access (TDMA) to ensure traffic balancing and efficient channel use. This protocol achieves significant savings in power by employing a sleep-wake strategy wherein the nodes that are not communicating are put into a doze mode.

**Figure 2:**
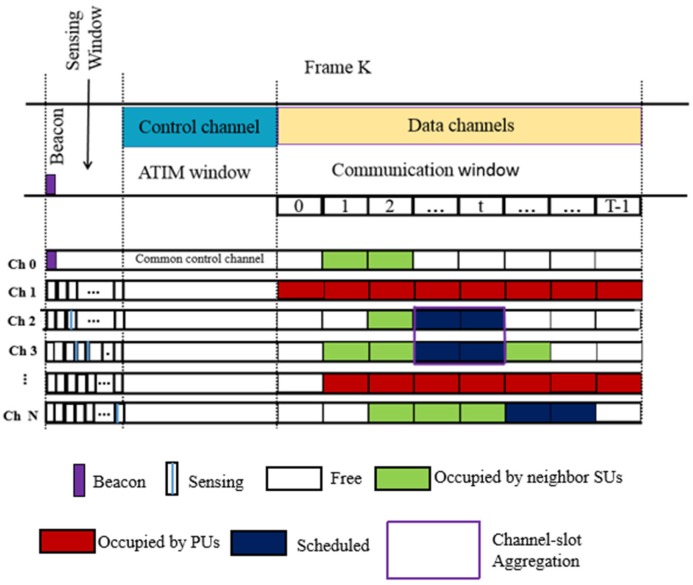
Frame structure of the energy-efficient cognitive radio medium access control (MAC) protocol for battlefield communications. PUs, primary users; SUs, secondary users; ATIM, ad hoc traffic indication message.

More importantly, frame length varies dynamically as per the number of nodes to achieve an improved network performance. The protocol provides strong criteria for determining how each of the secondary users chooses the appropriate channel at the appropriate time. It is a multichannel MAC protocol with dynamic channel selection making sure that communication among primary users is not hindered. This is achieved by dividing the system time into fixed time intervals, as shown in Figure 2. Each time window is allocated for a certain purpose, and this process has been rightly named as the channel-slot aggregation technique. A communication segment is allocated for each channel and time slot, denoted by the pair (l, t), where l is the channel and t is the time slot. A communication segment can be occupied, free or tentatively assigned based on the status of the channel involved (whether it is in use, free or is likely to be used in the next segment).

In fact, the proposed MAC structure consists of four main windows, namely the beacon window, the sensing window, the AITM window, and the communication window. A periodic beacon signal is broadcasted during the beacon window to ensure synchronization of all CR users. Moreover, during the sensing window, a thorough channel sensing process is performed in order to survey the channels and find spectrum opportunities. During an AITM window, all CR users tune to their radio interfaces and exchange communication control messages for resource allocation. Finally, during the communication window, CR users perform communication through the selected channels.

The beauty of this diversity technique lies in the fact that the CR nodes set the upper-bounded transmit power for each available channel based on their characteristics, ultimately contributing to energy efficiency. Additionally, it contributes in elimination contention among nodes and in decomposing traffic among multiple channels.

### 4.2. Energy-Efficient Non-Overlapping Channel (ENC) MAC for Cognitive Radio Enabled Sensor Networks

**Figure 3:**
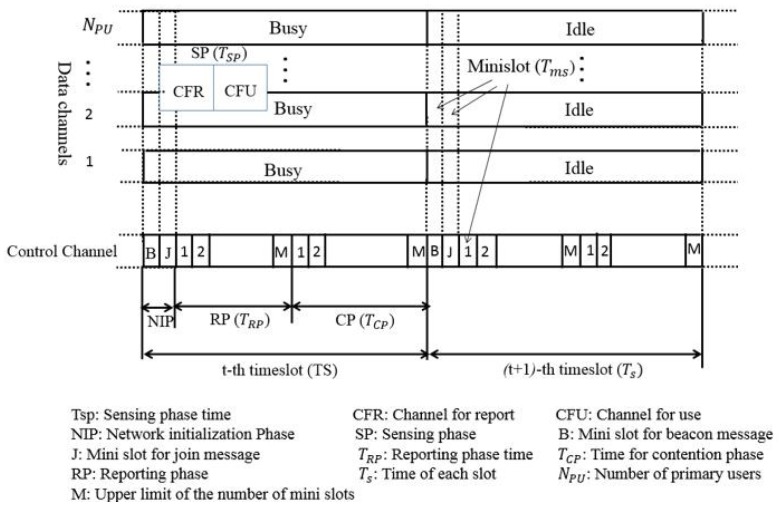
Operation of the ENC MAC protocol.

The ENC-MAC protocol uses two half-duplex transceivers; one is for control messages and the other one is for data [37]. In Figure 3, a complete time structure of this protocol is presented.

The operation of the ENC-MAC protocol can be explained in four distinct phases: The initialization phase, the sensing phase, the reporting phase, and the contention and data transmission phase.

During the initialization phase, the synchronization of a new SU is done by using a beacon message. The beacon message can be obtained from the existing master SU. If there is no master SU, then the new SU becomes the master SU and sends its beacon message to the other nodes along with a timestamp. Then the SU node competes for control of the channel to join during a J mini slot, which follows an 802.11 DCF back-off mechanism. After gaining access to the control channel, the master SU broadcasts a message on it using a J mini slot. The channel for report (CFR) is known to the SU and records the SUs identifier list. The SU index is maintained by listening to the exchange of join messages and during the reporting phase. In case of failure of the master SU, the SU having the lowest index value will act as a master SU and transmit beacon messages to other nodes.

During the reporting phase, the nodes cooperate by sharing information obtained by sensing about the states of channels. This information is conveyed in the report messages, which contains three fields: Index of SU (to identify the SU), CFR (for reporting on the data channel of that SU) and result (either idle or busy). The SU, after receiving the report message, updates the ACL.

The contention phase involves the channel reservation process, which also deals with control message collisions. For this purpose, there is an exchange of request-to-send (RTS) and clear-to-send (CTS) messages with a specified back off time (the same one as in the 802.11 DCF protocol). This process is carried out at the beginning of each slot. However, a modification is done on these messages by adding the CFU field. This CFU field facilitates the reservation of the CFU between the sender and the receiver. The other nodes overhear the RTS/CTS exchange and update their channel vectors accordingly to avoid multichannel hidden problem. Then, when sensing during the second mini slot of the following timeslot of the sensing phase, the winner SU senses the channel for use (CFU) in order to guarantee that the channel will still be available in the next slot.

### 4.3. Cognitive Radio Based (CRB) MAC: A Receiver-Based MAC Protocol for Cognitive Radio Equipped Smart Grid Sensor Networks 

This is a receiver-based MAC protocol, which employs preamble sampling [38]. The CRSN nodes perform asynchronous low-power listening, and they are capable of scheduling their own sleep/active cycle individually. This ensures significant amount of energy wastage in idle mode. In addition, due to asynchronous duty cycle of the nodes, the need of extra beaconing for the synchronization is eliminated.

**Figure 4:**
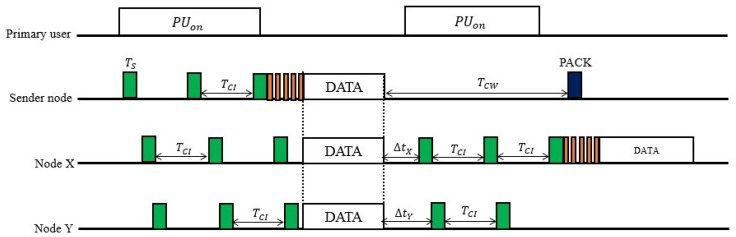
Operation of the CRB MAC protocol.

The operation of the CRB-MAC is shown in Figure 4. The sender node wants to send the data to the sink node. Node X and node Y are the contenders for the forwarding process. Whenever the sender node has data to send, it performs sensing operation for the channel. If there is presence of primary user in that particular time, it goes to sleep mode until the checking interval time ($T_{CI}$) expires. When the spectrum is found to be idle, the sender node transmits the preamble followed by the actual data. It is assumed that node X and node Y both are in the transmission range of the sender node in Figure 4. The micro frame of the preamble contains the information about the identification of the neighboring nodes in order to distinguish whether the transmission is a primary user transmission or the sensor node transmission.

Unlike sender-based MAC protocol, the number of retransmissions is significantly reduced as there will be multiple receivers to forward the data. The sensing time is also made adaptive according the information of the miss detection event. This mechanism mitigates the energy consumption for sensing, as well as improves the network performance as more time will be available for the actual data transfer. Moreover, the forwarding node is selected as the node having shortest distance from the sink node.

The nodes X and Y set the timers ∆X (relative to the distance of the node X from the sink node) and ∆Y (relative to the distance of the node X from the sink node), respectively, after receiving the data from the sender. The shortest path is considered to be the path through the node having lower value of this relative timer. Just after the time expires, nodes perform spectrum sensing. The sensing result may provide one of the following three conditions: Occupied by PU, sensor node transmission and channel is idle.

In the first condition (the channel is occupied by PU), the node goes to sleep mode for duration of checking interval $\left(T_{CI}\right)$. In the second condition (sensor node transmission), the node determines whether it should discard its own data or transmit it, which is determined by checking the sequence number of the transmitted data. The data packet is discarded if the sequence number of the transmitted data resembles with its own data packet. Finally, in the third condition (channel is idle), the node transmits the data packet as it is deemed to be a winner node. In Figure 4, node X is the winner node and it forwards the data packet.

However, when there is no successful transmission during contention window ($T_{CW}$), the sender node has to retransmit the data packet. The information of the successful or unsuccessful transmission is obtained by passive acknowledgement (PACK) according to the sensing result just before the duration contention window expires.

### 4.4. A Cluster-Based Energy-Efcient MAC Protocol for Multi-Hop Cognitive Radio Sensor Networks

The cluster-based energy-efficient MAC protocol named KoN-MAC was designed to allow nodes in multi-hop networks to select interference free channels dynamically [39]. The main concept of KoN-MAC is that a node can determine available channels just from sensing a subset of the available channels rather than sensing all the channels. This subset of channels is the polled-channel set.

Within the cluster of sensor nodes, a certain node will be selected as the cluster head (CH), and the gateway nodes (CG) will conduct communication between adjacent nodes. In addition, the remaining nodes are considered as cluster members (CM).

**Figure 5:**
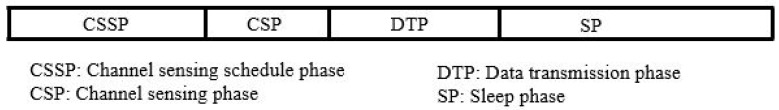
Superframe structure of the cluster-based energy-efficient MAC.

This cluster-based MAC Protocol, being a scheduled-based MAC-Protocol, uses four separate phases that comprise the superframe interval: the channel sensing schedule phase (CSSP), channel schedule phase (CSP), data transmission phase (DTP), and sleep phase (SP). These phases are shown in Figure 5.

The CSSP has two kinds of slots: Transmission slots and channel-sensing slots. The CH uses the transmission slot to transmit its own channel weight table to the rest of the nodes of the cluster. On the other hand, the channel sensing slots are used for cooperative sensing. Therefore, in each cluster, the CH holds the results sensed by the nodes and cooperative sensing is achieved using a data fusion method. On the other hand, the CSP is the phase in which channel allocation for the cluster member nodes of a cluster is carried out. Data transmission slots are also allocated in this phase. During the DTP phase, the members of a cluster transmit data in their assigned slot.

Now, in this section, the challenges related to multichannel access, multi-hop networks and cognitive radio technology will be clearly explained, and an idea for overcoming them, proposed by the author, is presented. The main issues with multichannel access are the traditional hidden and exposed terminal problems combined with the multi-channel hidden terminal problem. This is simply because a single half-duplex transceiver in a sensor node can either transmit or receive the packet at a given time on the control channel or the data channel. In addition, the performance of any wireless network decreases as the number of nodes increases. Finally, the challenge of cooperative sensing, information of PUs, dynamic channel selection and channel switching mechanisms also arise when considering cognitive radio technology. Aside from this, the KoN-MAC protocol also deals with the sleep/active mechanism for saving energy in each node.

According to the author, two-hop neighbor nodes are responsible for the multi-channel hidden terminal problem (which occurs when the same channel is selected by two neighboring nodes). While in this cluster-based structure, two-hop neighbors may be within the same cluster or in adjacent clusters. If they are within the same cluster, the CH schedules communication. If they are in adjacent clusters, they tend to select different channels and hence the probability of having multi-channel hidden terminal problems decreases.

Likewise, to enhance channel selection accuracy of channel, and that SUs have different probabilities to use different channels in the presence of PUs, the concept of channel weight is used. Channel weight is used to distinguish the channels from each other and ensures that nodes select the best channel based on the information available on channel weight. Every node will maintain its own channel weight table, which consists of different states (i.e., idle, busy, communication, and collision). Nodes will update their channel weight table immediately after sensing. A channel is idle if the SUs find the channel available for access, whereas it is considered as busy if the SUs detect the presence of PUs in that particular channel. These channel states will be determined in the CSSP, whereas collisions and communication happen in the DTP. Successful transmission of data on a channel by SUs is referred to as communication and the arrival of PUs or SUs while an SU is transmitting is considered a collision.

### 4.5. Energy-Efcient MAC Protocol for IEEE 802.11-Based Cognitive Radio Networks

The 802.11-based cognitive radio network (CRN) MAC Protocol was proposed for single-hop ad-hoc cognitive networks [40]. The limited time for SUs transmission, as well as the frequent contention among SUs causes not only an energy overuse, but also increases power consumption at the destination node. Considering this, the proposed MAC protocol uses a single sender node to transmit a sequence of packets to its intended receiver in the active state until the next spectrum-sensing period begins, while keeping other nodes in the sleep state and conducting spectrum sensing over short periods. Unlike the conventional FIFO (first-in, first-out) transmission policy, packets are ordered in the queue and manipulated in such a way that improves energy efficiency. Once the sender node occupies the channel and transmits its packets to multiple destinations, only the sender and the receiver stay in the awake state while other nodes just switch to sleep mode in order to save energy. The backlogged packets in the sender node ensure a higher energy efficiency and data throughput as the number of destination nodes decreases.

**Figure 6:**
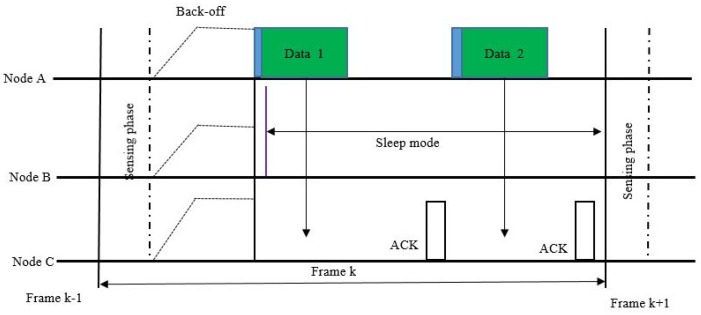
Example of operation of the energy-efficient MAC Protocol for IEEE 802.11-based cognitive radio networks (CRNs).

Shown in Figure 6 is an example of operation of the proposed MAC protocol, which can be summarized in three steps. First, the node having the smallest back-off number is selected after the back-off mechanism is used in each node once the channel is confirmed to be free from PUs. Then, the transmission order is determined for the queued candidate packets by the sender. The sender attaches a header containing transmission information in the first packet to be transmitted. Finally, the set of intended receivers stays active to receive the data packets whereas other nodes switch to sleep mode. In Figure 6, it can be seen that during the duration of one frame there is communication between nodes A and C, and they are active. Meanwhile, node B notices that the channel is already occupied and, since it is not the intended receiver of the message, switches to sleep mode in order to save energy.

### 4.6. Cognitive Radio Wireless Sensor Networks (CR-WSN) MAC: An Energy-Efcient and Spectrum-Aware MAC Protocol for CRSNs 

This is yet another energy-efficient MAC protocol for CRSN [41]. Since there is no synchronization overhead, it has been claimed that this MAC protocol is more energy-efficient than synchronized ones. In this protocol, the coverage area of PUs is assumed to be smaller than that of SUs, and that SUs use a dedicated common control channel for the exchange of channel reservation data. The preamble packets sent on the CCC are short and multiple in number. Since this short preamble contains the address of the destination node and the channel sensing results, it enables non-destination nodes to go into sleep mode after hearing only the first preamble, rather than having to wait for an extended preamble.

**Figure 7:**
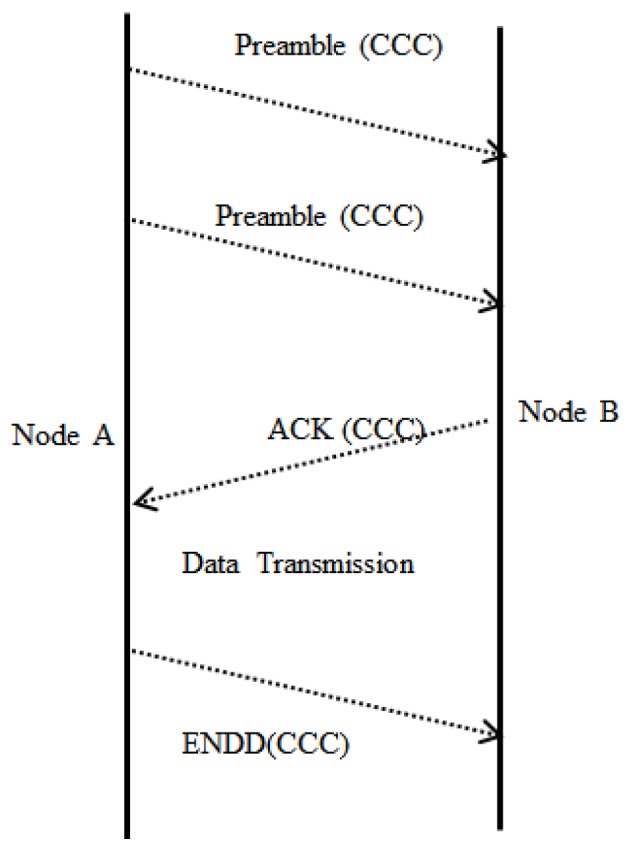
Operation of the CR-WSN MAC protocol. CCC, common control channel; ACK, acknowledgement; ENDD, end of data.

**Figure 8:**
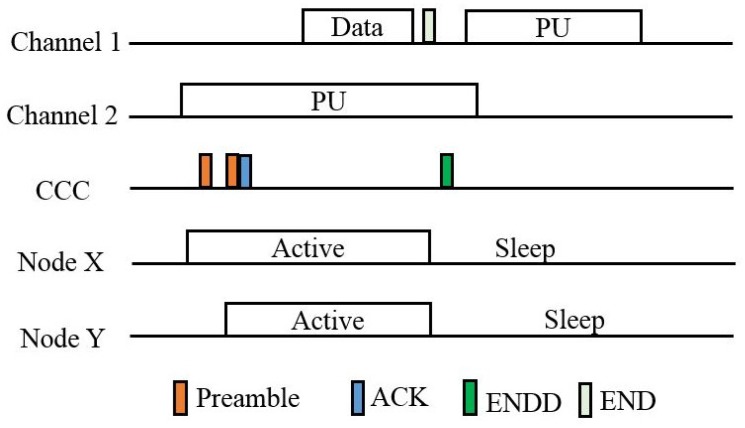
Timing diagram of the CR-WSN MAC protocol. ENDD, end of data.

Each sensor node follows a sleep/active cycle and senses all the data channels, thus storing the status of each channel in a vector at the start of each cycle. Each node listens to the CCC while active and, if a data transmission request is not found, switches to sleep mode. Otherwise, after receiving data transmission request, the receiving node resets its timer and sends an acknowledgement (ACK) message (which contains the ID of the data channel to be used) on the CCC to ensure that data transmission is carried out on the selected channel, as shown in Figure 7. Since the transmission process on a data channel is considered to be the combination of packet transmission and channel sensing intervals, a periodic sensing approach is used in order to mitigate interference between PUs and SUs. The senders and receivers discard a packet if the presence of a PU is detected and leave that particular data channel free from SU transmissions. Finally, an end of data (ENDD) is broadcasted on the CCC so that other nodes listening to the CCC update their channel state vector accordingly. The ENDD contains identifiers containing information about the latest sensing results obtained by the pair of nodes taking part in the transmission on that data channel. Figure 8 depicts the communication flow described above.

### 4.7. A Multi-Constrained QoS-Aware MAC Protocol for Cluster-Based CRSNs 

This protocol focuses not only on energy consumption, but also on various QoS constrains of data packets, such as reliability and delay [42]. To realize such a delay-and-reliability-aware traffic, separate slots are assigned, namely the guaranteed time slots (GTS). A dynamic data and backup channel assignment mechanism reduces the number of retransmission, which, in turn, saves significant amount of energy. The GTSs and post-contention periods are dynamic, which also helps in saving energy if there is less traffic generated by the sensor nodes.

**Figure 9:**
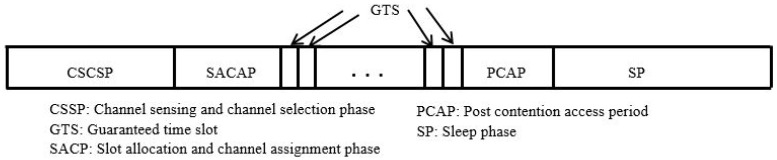
Superframe structure of the multi-constrained QoS-aware MAC protocol.

The operation of this MAC protocol can be explained in four different phases, as shown above in Figure 9. These phases are the cooperative sensing channel selection phase (CSCSP), slot allocation and channel assignment phase (SACAP), DTP, and SP. The DTP is further divided into GTSs and post contention access period (PCAP).

Another phase is the SACAP, in which GTSs are allocated for traffic from the nodes, which are delay constrained and have a shorter lifetime. This is followed by the selection of the data channel and GTS backup channels for each of the GTSs. Then, the cluster head assigns channels to each node that requested transmission. Channels are assigned in such a way that the channel having a high channel weight will get multiple slots whereas low ranking channels will be assigned only a single slot. For best-effort traffic, no slots are allocated and it employs CSMA/CA mechanisms to transmit packets during the PCAP phase.

The DTP phase is further divided into GTSs and the PCAP where best effort transmissions of best-effort data are carried out using a random back-off mechanism. The remaining lifetime of the packet is regarded as a decision making parameter for transmission order. Packets having the lowest lifetime will be transmitted first, and so on. Packets received from cluster members are now collected in the cluster head and are transmitted to the next CH using a CSMA/CA-based medium access mechanism, until they reach the sink. Finally, during the sleep phase, the transceiver switches to sleep mode in order to save energy.

The proposed multi-constrained QoS aware MAC protocol makes differently constrained QoS-aware traffic possible. Moreover, since the best channels are selected, data retransmission rate is also reduced, thus reducing power consumption. In addition, calculations using the subset of available channels reduces the sensing overhead, which also helps in saving energy. However, additional computational overhead is required for defining and processing various parameters.

### 4.8. Cognitive Adaptive (CA) MAC: Cognitive Adaptive Medium Access Control in CRSNs

The Cognitive Adaptive Medium Access Control protocol has been proposed in literature, which follows an on-demand spectrum sensing mechanism [43]. This protocol adapts the spatial correlation of the nodes so that the spectrum correlating (CN) nodes and the spectrum representative (SR) nodes are aligned. The SR node are the ones that take part in sensing the spectrum. The operation of this MAC protocol is divided into three phases, namely, the spectrum measurement phase, the channel contention phase and the transmission phase.

During the spectrum measurement phase, when a data transmission request reaches a node (either by an event or a forwarding request), that particular node first checks the neighboring table to see whether there are SR nodes. If there are, the node becomes a CN node and then selects an SR node. Otherwise, if no SR node is found on the neighboring table, the node acts as an SR node and it performs the spectrum sensing. A hello beacon is transmitted after the periodic hello interval in order to update the nodes status in the table.

On the other hand, if the node satisfies the conditions to be a CN node, it sends a request for spectrum information over the common control channel to the selected SR node on its neighboring table. However, there is a chance that the time interval for receiving back an acknowledgement from the specified SR node may expire. In this case, the CN node tries to find another SR node from the neighboring table. If there are no more SR nodes to probe, then the CN node becomes an SR node and starts the spectrum measurement process again.

During the channel contention phase, the CN node sends a negotiation request with its available channels in order of priority over the common control channel to the receiver node. The receiver node does not have enough information at this point about the available channel list, so it switches to the spectrum management phase again and looks for available channels. An acknowledgement message is transmitted only to that sender node with a list of the preferred available channels the sender node then it switches to the receivers preferred data channel. If there are no available common channels, then the sender node switches into a new spectrum management phase again.

The data transmission phase is the one in which real data transmissions are carried out using a CSMA mechanism. The sender node has to confirm again whether the preferred channel is still appropriate for data transmission or not, and if the receivers preferred available channel is not suitable for the sender node, the sender nodes sends the preferred channel list to the receiver again through the receivers preferred channel. The receiving node then switches to the data channel suggested by the sender node for the real data transmission, and thus both the sender and the receiver nodes tune into the same data channel. In case of failure of transmission of this data, the nodes switch into the contention phase again and follow the steps accordingly.

The unique feature that is proposed in this study is that if the size of the data to be sent from the sensor node is small, then this data is sent with an RTS packet by a process of piggybacking. Likewise, the acknowledgement (ACK) packet is also piggybacked on the CTS. This mechanism is beneficial in terms of reducing transmission overhead for small data packets. However, it is not explained how to decide if the size of the packet from a node is small enough to piggyback.

Energy is saved during the sensing phase by adopting an adaptive sensing period. In order to find an efficient value for the sensing period (i.e., a decent balance between fast and fine sensing), channels are rewarded whenever successful data communication is carried out through them. Likewise, a penalty is given to a channel after every transmission failure occurs on said channel. The sensing overhead at the nodes is reduced by using the information of the spatial correlation of the nodes. Thus, the obtained result is shared to the neighboring nodes. Cooperative sensing, adaptive duty cycle, and on demand spectrum sensing are the main mechanisms that are responsible for saving energy in this protocol.

### 4.9. MAC Protocol for CR-WSN without a Dedicated Common Control Channel (DCCC)

This ad-hoc clustered CRSN MAC protocol does not use a dedicated common control channel for the transmission of the control signals [10]. In fact, the time of the licensed channels is divided into default slots.

**Figure 10:**
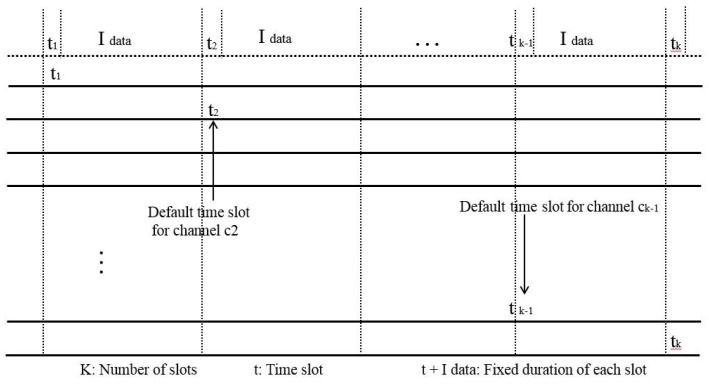
Channels with default time slots.

This protocol uses two half-duplex transceivers; one is the control transceiver while the other one is the data transceiver. The control transceiver of each sensor node rendezvous during the channels default time, which is used for exchanging control message and check the status of neighboring nodes. Channel time is divided into default time slots of length ‘t+I Data’. A representation of ‘k’ channels with a fixed-duration is shown in Figure 10.

**Figure 11:**
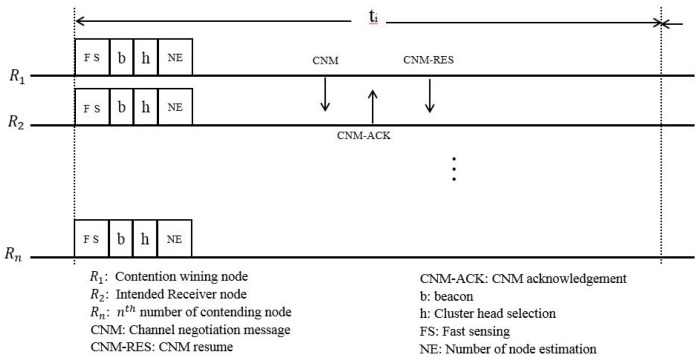
Channel negotiation in the default slot.

The operation of this MAC protocol begins with a fast sensing process, the result of which is recorded in a channel status table. The channel status table contains three categories of statuses, according to different channel situations: When an incumbent user is active on the channel, when channel is in an idle state and when the status of the channel is uncertain. Then, the nodes tune their control transceiver to the default slot of the channels. If no incumbent user activity is detected after sensing, the node which was selected after the contention process ($R_{1}$) sends a channel negotiation message (CNM) to the intended receiver node ($R_{2}$), which is received through a selected common available channel, as shown in Figure 11. A CNM resume (CNM-RES) message is sent to the receiver node while the others update their channel status table. On the other hand, if the activity of an incumbent user is found, the nodes skip the current default time slot and wait for the next one for another negotiation round.

Then preferred channel list (PCL) in the sensor node is estimated. This list consists of a ranking of the channels using an exponentially weighted moving average filter. For the same channels, the priority detected for different sensor nodes may vary depending on geographical location and time. Sensor nodes select the common available channel having the highest priority, and then both the sender and receiver nodes hop to that channel. Data transmission then commences on that particular channel. For energy saving purposes, the control transceivers also switch to a doze state when a PU is detected and according to the estimated density nodes.

**Figure 12:**
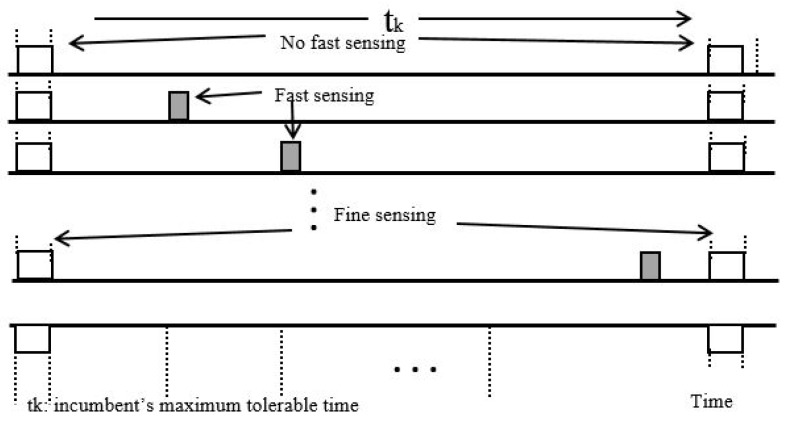
Approximate illustration of fast and fine sensing.

Figure 12 shows how channel sensing is adaptive. Fast sensing is initially carried out, but since this is not enough to identify whether a channel is either in a busy or idle condition, three possible status are determined: Idle, busy, and uncertain. After performing fast sensing for up to the maximum incumbent user interference tolerable time (t_k_), fine sensing is carried out. This reduces the number of false alarms and enhances the QoS.

### 4.10. Opportunistic Channel Selection (OCS) MAC Protocol for Cognitive Radio Ad Hoc Sensor Networks

The opportunistic channel selection MAC protocol for cognitive radio ad hoc sensor networks in the internet of things (IoT) does not use CCC to exchange control information and the sensor nodes are equipped with two radio interfaces. This protocol is mainly focused on the opportunistic channel switching mechanism to ensure better energy efficiency, goodput, and channel utilization [44]. The high energy efficiency of the protocol is acquired because more successful transmissions are achieved due to the opportunistic channel selection scheme.

The operational mechanism in the proposed MAC protocol is based on the legacy IEEE 802.11 with some modification on the RTS frame structure. An additional field for the information about the best channel preferred by the sender node is introduced. The channel selection mechanism is split in two phases. In the first phase, the data frames are transmitted in a sequential order. The number of data frame transmissions is measured. In the second phase, the channel through which the largest number of data frame transmissions occurs is given the highest priority for the next transmission. This information is included in the modified RTS frame structure.

All the sensor nodes maintain the information of the channels in a channel lookup table. When a sender has the data to send, it sends the RTS packet to the receiver. The RTS packet contains the information about the best channel. The neighboring nodes overhearing the RTS packet update their lookup table and do not transmit on that specified channel. The sender after receiving the CTS packet maintains the channel lookup table. After the negotiation on that specified channel by the sender, the radio interface switches to that channel and the data transmission occurs.

## 5. Comparison of Energy-Efficient MAC Protocols for CRSNs

In this section, the different energy-efficient MAC protocols designed for CRSNs presented are compared against each other in terms of network configuration, common control channel, spectrum sensing technique, channel access mechanism, synchronization, advantages, and disadvantages. Comparison results are summarized in Table 1.

In Reference [38], the receiver with the highest transmission energy efficiency is selected by the auction mechanism and the receiver node whose transmission energy required is the least is selected as a winner. Then, data is forwarded to the next hop. Thus, node which has the shortest distance from the sink node takes part in communication. However, we cannot guarantee that the selected path is the energy-efficient path because the shortest path does not mean the energy efficient path. The traffic is also classified whether it is delay sensitive or not. For delay sensitive traffic the priority is given for the transmission. Another feature of this protocol is that it uses preamble sampling to conserve energy during idle listening by making unintended nodes switch into sleep mode. Since this is a receiver-initiated protocol, the sender node does not need to wake up for a long time until it finds that the receiver is awake.

The protocol presented in Reference [40] uses a single sender node to transmit a sequence of packets to the multiple receiver nodes that are in an active state until the next sensing period begins. Since limited time for a sender to transmit data and frequent contention consumes more energy, addressing these issues can save the energy. After the channel for communication gets occupied by multiple intended receivers, the unintended nodes go to sleep mode. Only four types of power consumption are discussed here: Idle, transmitting, receiving, and in sleep mode. However, the power consumed for spectrum sensing and mode switching is ignored. Total power consumption is calculated as the ratio of the sum of the four aforementioned consumptions over the duration of one frame multiplied by the total duration of all frames.

In Reference [41], an asynchronous CRSN MAC protocol is proposed in which the duty cycle of the nodes is made adaptive. This eliminates synchronization overhead by conserving energy. Here, a burst of preamble packets is sent to the destination node by the sender node through the dedicated channel. The preamble packet includes destination address and the available channels. When the receiver receives the first packet, it sends an acknowledgement to stop further packet to the sender nodes and, at the same time, the neighboring nodes listening to the control channel go to sleep mode. However, this protocol does not address the quality of the available channels, which increases the possibility of collisions.

The CRSN nodes in Reference [36] set the upper-bounded transmit power for each available channel based on their characteristics, ultimately contributing to energy efficiency. Additionally, it contributes in elimination contention among nodes and in decomposing traffic among multiple channels. For large networks, however, it suffers from synchronization overhead. In CRSNs, the channel aggregation technique applied may be more difficult to maintain, due to the variable channel conditions.

In Reference [43], the cooperative sensing is deployed in such a way that each SU maintains the information of the channels regarding activity of the PU that reduces the collision probability. Moreover, only two mini slots in the sensing phase is enough by reducing the sensing time and consequently the energy consumption. However, selecting the master node to send the beacon message and frequent beacon packet transmission among the sensor nodes causes an extra overhead for this protocol.

The cluster-based MAC protocol for CRSN is studied in References [39,42]. In Reference [42], the available channels are ranked according to their weight for the effective spectrum hand-off. The weight includes the status of the channel during sensing whether it is idle or busy, the transmission is successful or not, and is there any collision on a particular channel or not. The main concept of this protocol is to sense only some of the available spectrum channel rather than sensing all of them, which reduces sensing time and hence conserve energy. The concept of back-up channel is also used. Reference [42] follows the same operation for the sensing by implying the optimal subset of the channels for sensing. It also assigns the back-up channels. Here, the data traffic is classified in four different categories according to the traffic different application and the channel allocation is also carried out based on the type of this traffic type. However, both aforementioned MAC protocols for CRSNs are schedule-based protocol that involves in sensing and cluster forming operation even if there is no data to send or very less traffic of the data. In addition, by sensing only the subset of the available channels rather than sensing all channels, it does not seem convincing idea to rank all the available channels.

The protocol proposed in Reference [10] does not use a dedicated channel for the exchange of control messages. Instead, it uses two transceivers; one for control messages and one for data. The control transceivers from the sensor nodes rendezvous during the channels default time, which is used for exchanging control messages and snooping the neighboring nodes status. Another unique feature of this protocol is that, even though it uses two radios, the control radio also switches to a sleep/doze state when a PU is detected, in order to save energy. However, this design is complex as the control radio that is used to exchange control packets also goes to doze mode independently along with the traffic radio when there is completion of control packet transmission. Higher contention is anticipated when there is a large number of nodes, as it does not use the dedicated control channel for the control packets.

The MAC protocol proposed in Reference [44] prioritizes the available channels in terms of the number of transmitted frames through the particular channel. The information about the best channel preferred by the sender is included in the RTS packet. However, there is no information about the receiver’s preferred channel information in the CTS packet. Therefore, when the best channel preferred by the sender is not available in the receiver, then it needs to spend more time for the negotiation of the channels, which may require the sensing algorithm to run several times.

The MAC protocols proposed in References [38,40,41,43] are appropriate for large networks, especially for event monitoring application areas in which there is less traffic load. Such application areas may be surveillance, smart grids, etc. This is because of the low synchronization overhead and the sleep/active mechanism of the transceivers in these protocols. On the other hand, References [10,36,44] are more suitable for application areas with high traffic load and in areas where more reliable transmission is required, like in relatively smaller networks, such as those for multimedia applications. However, the protocol proposed in Reference [39] is better for application areas that have medium levels of traffic from the node, while the protocol presented in Reference [42] is suitable for various QoS-constrained application areas.

The proposed MAC protocols for CRSNs exploit the popular design technique of WSNs in which the operation mode of the nodes switches to sleep mode in order to save the energy during ideal listening. Furthermore, these MAC protocols for CRSNs focus on saving energy by selecting an optimal sensing period by using different techniques and considering the hidden terminal problem, false alarm probability, missed detections, etc. In addition, the focus is also on selecting reliable data and backup channels in order to avoid energy wastage, due to collision and retransmission. Although using non-dedicated channels seems to be a not very effective solution for large networks, in Reference [10] an attempt of designing such a MAC protocol is presented and promising energy efficiency and throughput could be obtained for small area networks.

## 6. Open Issues and Challenges

CRSNs present several challenges when designing an appropriate protocol. The analysis of the aforementioned MAC protocols presented in Section 3 may significantly help in the design of appropriate MAC protocols by addressing their shortcomings, as well as considering their different modes of application. In addition, in the future, researchers should tackle a substantial amount of challenges in order to design better CRSNs to ensure their implementation in a real environment. Some of these challenges can be described as follows:

### 6.1. Cross-Layer Design

Generally, in TCP/IP networks, each layer is designed in such a way that there is only a limited interface that connects it to the immediate upper or lower layer. Cross-layering is a technique in which certain parameters from two or more layers can be borrowed or merged as per the requirements of the specific application area [7], and this approach can increase power efficiency in WSN communications [45]. However, these approaches used in conventional WSNs cannot be directly adapted for CRSNs. There have also been some cross-layer designs for cognitive radio, which mainly focuses on increasing throughput and delay while energy efficiency is not considered. To the best of our knowledge, until now, there are not a significant number of cross-layer designs for MAC protocols in existing CRSNs.

### 6.2. Coexistance with Other Technologies

It is obvious that the signal strength of CRSNs are relatively lower than that of the other existing high power communication systems, such as cellular communication, wireless local area network (WLAN), etc. Thus, there will be severe interference, due to such wireless systems when they coexist together. In order to achieve better energy efficiency along with the network performance, the channels having less interference are to be selected. In the literature [46], an efficient access mechanism is discussed under the coexistence of the high power WLAN systems.

### 6.3. Fault Tolerance

There is a chance of failure in any link or node due to various reasons, such as malfunctioning of hardware or software, natural disasters etc. Therefore, the MAC protocol for CRSNs should be designed in such a way that meets a certain level of fault tolerance so that communication will not be disturbed even if some of the nodes are not operational. This is one of the objectives of any network communication system, and is particularly important for CRSNs; they have the added responsibility of protecting the PUs. Thus, the design of CRSN MAC protocols should take the fault tolerance levels of the network into consideration.

### 6.4. Quality of Service (QoS)

CRSNs require a good QoS not only in terms of bandwidth, delay, jitter and reliability, but also in terms of protection of PUs from interference caused by SUs, ensuring access to the incumbent spectrum. The prediction of the arrival of Pus on a particular channel is a difficult task, and may lead to serious consequences. Conducting research on the behavior of PUs in a network for mitigating missed detections of the primary signal and false alarms is mandatory.

### 6.5. Clustering

In CRSNs where a large number of sensor nodes are present, clustering is a valid approach to achieve lower energy consumption levels and less communication overhead along with improved scalability. There are some clustering techniques in existence, such as static clustering, dynamic clustering, single-hop and multiple-hop clustering, homogenous clustering and heterogeneous clustering. However, a thorough analysis of the clustering scheme also considering the cognitive radio properties of CRSNs is indispensable.

### 6.6. Energy-Efficient Sensing Techniques

The main function of CRSNs is to detect vacant spectrum channels. Moreover, efficient sensing is crucial for observing the status of such channels during operation for good decision making. The parameters used in the sensing tasks significantly impact energy consumption levels during communication, along with other communication performance values, such as delay, throughput, etc. Sensing methods can be described by three main characteristics: Sensing technique used for the transmitted signal, sensing duration, and sensing cooperation. For the spatially distributed spectrum sensors, the cooperative sensing technique ensures better detection performance for homogenous environment as compared to the non-cooperated sensing technique. On the other hand, for spatiotemporal diverse heterogeneous networks, the performance metrics to guide the design of joint spatiotemporal spectrum sensing are suggested in Reference [47].

### 6.7. Channel Assignment

CRSN channels are dynamic in nature (unlike WSN channels), and the CRSN bears the responsibility of protecting the primary users communication. These two characteristics demand a suitable channel assignment strategy. Channel assignment strategies can be mainly categorized into three types: Prediction model, database information, and periodic sensing results.

### 6.8. Number of Radios

For an efficient design of a MAC protocol for CRSNs, the number of radios in the sensor node is an important consideration. The whole design process is affected by this number. The implementation of single-radio nodes is quite popular in conventional WSNs, where the main objective is saving energy. In addition, lowering costs and hardware design complexity results in more promising protocols for sensor networks. Two or more radios can be used to make the design of MAC protocols simpler because of the significant features they enable, particularly tuning and accessing different channels simultaneously. These capabilities help the CRSN avoid collisions with PUs and the multichannel hidden terminal problem.

### 6.9. Number of Transmission Channels

The selection of the number of transmission channels between pairing nodes in CRSNs determines the design of the MAC protocol. Data can be transmitted using a single channel or multiple channels. In a single-channel scheme, the communicating CRSN pair uses a single channel for data transmission, which eliminates the requirement of reserving more spectrum resources, which in turn makes transmission less complex. In multichannel schemes, two or more channels are used for data transmission between pairing nodes. A scheme that uses multichannel transmissions with a single radio is better in terms of the hardware costs and energy requirements however the design process becomes more complex. Such a scheme can be implemented using either the channel aggregation or the channel bonding techniques [48].

### 6.10. Common Control Channel Selection

The common control channel plays a pivotal role in the design of efficient MAC protocols. In CRSNs, a larger number of exchanges of control messages are required than in conventional multichannel WSNs, in which they are sent over a common control channel. In general, control channels are used for network initialization, nodes negotiation, reporting of available channels and neighbors lists, etc. [49]. There are two categories of common control channels, which are based on the way the channel for control messages is selected. In a static scheme, one of the channels is always available for control message exchange among the nodes, in which no PU arrives. On the other hand, the common control channel can be selected dynamically; this is referred to as a dynamic scheme. Even though the dedicated channel approach is vulnerable to channel saturation and denial-of-service (DoS) attacks, a well-designed dedicated scheme can enhance the aggregated bandwidth and reduces packet delay [50].

### 6.11. Energy Harvesting

Energy harvesting technology is intriguing research topics as it enhances the network lifetime of the nodes by supplying the necessary power from various sources. An additional unit for charging nodes is introduced as an energy harvesting technique, which exploits the energy generated from the different sources, such as solar energy, vibrational energy, wind, electromagnetic radiation, etc. A radio frequency (RF) harvesting mechanism that charges the sensor node using the primary user’s radio frequency has been discussed in References [51,52,53]. When using this mechanism, both spectrum access and charging from PUs cannot take place at the same time. The optimum period and operational mode should be designed considering the nature of the harvesting techniques to be used while designing a MAC protocol for CRSNs.

### 6.12. Security and Threats

Attacking CRSNs is quite difficult for attackers because they use a dynamic spectrum, and unlike conventional WSNs that use a fixed spectrum. Nevertheless, with introduction of cognitive radios, CRSNs may become more vulnerable to several attacks and threats that are inherited from cognitive radio technology. During channel negotiation between two nodes, three kinds of frames may exist, which have information about the free channel list (which indicates available channel at the transmitting node), channel selection (denoting receiving nodes selected channel) and the reservation of communication channels [20]. Therefore, DoS attacks may become common in a CRSN network, which would cause the common control channel to saturate. Similarly, greedy nodes may reserve the available channel for their own use rather than relaying data to the destination nodes. These two issues may incur in degradation of communication performance of the whole system and, obviously, higher energy consumption could be caused.

Another security issue is related to the sensing algorithm. Attackers may inject a fake signal whose characteristics resemble the licensed primary users signal. This is known as primary user emulation attack [54], and is more serious for network where an energy detecting algorithm for sensing is used. However, cyclo-stationary algorithms are also not free from this type of attack, even if they can detect the intrinsic characteristics of PU signals. The attacker could send signals having the same spectral characteristics as the PU. Mechanism to identify this issue is discussed in Reference [55] and some cryptographic security methods have been proposed in the literature [56]. The overhead of this cryptographic security algorithm, which leads to a higher energy consumption, should be analyzed wisely so that a satisfactory level of energy efficiency can be obtained at the sensor nodes.

### 6.13. Mobility

Although nodes and sinks are less mobile in CRSNs compared with other cognitive radio networks [21], there are some applications of sensor nodes embedded in unmanned aerial vehicles (UAVs), robots, etc. The sink node can be also mobile in some cases and stops or slower the speed in order to receive data where the sensors are densely deployed [57]. Similarly, there are several researches where a mobile UAV is used to gather data from WSNs. In UAVs, however, the mobility is generally three dimensional and the speed is much faster. This increases the complexity in the design of energy-efficient MAC protocols in such scenarios. Thus, considerations on mobility are indispensable in order to carry out better spectrum management and, particularly, spectrum sensing, spectrum decisions and spectrum handoffs at the MAC level. This is because the topology of the network changes with the movements of the elements of a CRSN. In addition, with this change, the algorithms designed for static networks may not be effective in terms of sensing accuracy and allocation processes.

## 7. Conclusions

In this paper, energy-efficient MAC protocols for CRSNs have been surveyed. After overviewing the major features of CRSNs, the energy efficiency in the design of MAC protocols for CRSNs has been discussed. Then, the existing energy-efficient MAC protocols for CRSNs are extensively reviewed and qualitatively compared. The open issues and research challenges have been discussed as well. Because energy efficiency and spectrum management are very important design considerations in CRSNs, a well-designed energy-efficient MAC protocol is highly required. In particular, the network architecture, the number of radios, the selection of appropriate transmission and control channels, efficient channel sensing and selection mechanism, and an appropriate resource allocation technique are major design issues for researchers and engineers. In CRSNs, since PUs should be free from any interference caused by SUs, it is highly necessary to pay more attention to false alarms and missed detections during spectrum sensing. However, there may be a trade-off between energy efficiency and QoS in the design of a MAC protocol for CRSNs. In addition, the application areas should be always taken into. This survey will be helpful for researchers and engineers aiming to develop an energy-efficient MAC protocol for CRSNs. On the other hand, it is absolutely true that it would be much better to present the results of numerical analysis and simulation in a quantitative manner. Because the reviewed protocols exhibit different network topologies and working mechanisms, the computer simulation is preferable compared to the numerical analysis and it will be our future work.

## Figures and Tables

**Table 1:** Comparison of energy-efficient MAC protocols for CRSNs.

MAC Protocol	Network Configuration	CCC	Spectrum Sensing Technique	Channel Access Mechanism	Synchronization	Advantages	Limitations
CRB MAC [38]	Ad-hoc	Not specified	Energy detection	Contention based	Asynchronous	Lower duty cycle Low-power listening is enough for asynchronous preamble packets Fewer retransmissions in lossy wireless environments	Longer preamble packet transmission that causes higher energy consumption
Cluster-based MAC [39]	Ad-hoc cluster	Dedicated	Not specified	TDMA	Synchronous	No hidden or exposed terminals Fewer retransmissions due to backup channel	Less QoS provisioning
802.11-based MAC [40]	Ad-hoc	Not specified	Not specified	Contention based	Asynchronous	Backlogging of packets in sender nodes	Only for single-hop topology
CR-WSN MAC [41]	Ad-hoc	Dedicated	Energy detection	Contention based	Asynchronous	No synchronization overhead for large networks	Receiver uncertainty problem Hidden terminal problem
Battlefield MAC [36]	Ad-hoc	Dedicated control channel	Not specified	TDMA	Synchronous	Each node can utilize a group of channel-slots	Synchronization overhead for big networks
ENC MAC [37]	Ad-hoc	Dedicated control channel	Not specified	Hybrid	Synchronous	Each node can utilize a group of channel slots	Requires two transceivers
CAMAC [43]	Ad-hoc	Dedicated	Energy detection	Contention based	Asynchronous	Low CSMA overhead for small data packets due to piggybacking	Long channel negotiation process Hidden-node problem
Multi-constrained QoS MAC [42]	Ad-hoc cluster	Dedicated	Not specified	Hybrid	Synchronous	QoS is promising for different types of traffic Reliable backup channel	Hidden and exposed terminal issues are not explained
MAC without DCCC [10]	Ad-hoc cluster	Non-dedicated	Energy detection	Hybrid	Synchronous	No cost overhead for control channel	Tight synchronization is mandatory Not suitable for large networks Requires two transceivers
OCS MAC [44]	Ad-hoc cluster	Non-dedicated	Not specified	Contention based	Asynchronous	No cost overhead for control channel	Not suitable for large networks Additional sensing may require.
